# A New Use for the Phonograph

**Published:** 1889-12

**Authors:** 


### A NEW USE FOR THE PHONOGRAPH.

Dr. Walker, with his characteristic enterprise, brought out the phonograph in a new *rôle* at the last meeting of the First District Dental Society, November 12. Dr. L. Ashley Faught (Philadelphia) had prepared a paper for the society on "Some Notes on Methods of Practice," which he consented to read before a few invited members of the profession in Philadelphia previous to reading it in New York, in order that the subject might be discussed into the phonograph and repeated at the New York meeting. The object being to add to the interest by introducing a novelty and at the same time conserving the interests of science by bringing the ideas of absent members of the profession into the meeting. It was a decided success; the reproduction of the voice was so perfect that no difficulty was experienced in recognizing the speaker. Mr. Edison's agent said in substance, in introducing the instrument, that,—

"Although some ten years have elapsed since Edison startled the world by inventing a machine that recorded sound and reproduced it at will, it is only within the past few months that this wonderful invention has been brought to such a degree of perfection as makes it of practical utility to the public at large. The original phonograph, while valuable in demonstrating the possibilities of human invention, and as opening up a field of research in acoustics previously unexplored, lacks the elements essential for practical use. The metallic and sometimes indistinct sound-waves that were emitted from the primitive tin foil, the lack of proper incidental mechanical appliances, the want of a suitable material for the impression plate, and numerous other defects, made the invention useful principally in the laboratory of the scientist, or in the museum of curiosities.

"These early defects have at last been all overcome, and the perfected phonograph and phonograph-graphophone, for the multitudinous purposes for which they can be used, are as practical as the type-writer or the telephone in their respective spheres. By them the slightest shades and variations of the human voice are registered and reproduced with absolute accuracy. Music, whether vocal or instrumental, solo or multiple, in all its rhythm, melody and intonation, the lowest as well as the highest notes,—in a word, all sounds of every kind and character, may be treasured up in these extraordinary machines and reproduced, not once, but thousands of times, and may be mechanically duplicated and multiplied to any extent. The utility of this invention at this early day can scarcely be estimated. The uses of an instrument with such manifold functions would seem to be circumscribed only by the uses subserved by the property of sound itself."

The instrument was so arranged that all in the room could very distinctly hear most of what had been said into it at the preliminary meeting in Philadelphia. And thus were added to the discussion on the paper by the New York members and visitors the remarks of Professors Truman, Guilford, and Laplace, and Drs. Head, Kingsbury, and Roberts, of Philadelphia, who were unable to attend the New York meeting. Previous to the introduction of the discussions, however, a few typical specimens of its power were shown. The music of a martial band had been received upon a cylinder, and was reproduced with great distinctness even to the notes of a bugle-call in the distance. A minstrel quartette had sung into the phonograph a negro melody, into which had been introduced the sound of a steamboat whistle and the ringing of her bells as she left the landing. These were reproduced with such vividness as to almost carry one "away down among the cornfields." These and other pieces were presented to prepare the society for the demonstration that followed. This was considered necessary, because the Philadelphia members had never had any previous experience in speaking into the instrument. Regarding this unacquaintance, Dr. Truman facetiously expressed himself by saying that while it gave him great pleasure to thus converse with the members of the First District Society in the guise of a "spirit," yet he was not very well acquainted with the "medium," but hoped that it would do him justice. In consequence of this unfamiliarity with the instrument the remarks of some of the gentlemen were audible only to a few in the audience, but were distinctly reproduced by means of the ear-tubes.

The meeting was a decided success. There were fully one hundred and fifty in attendance, and all were delighted with the experiment, for experiment it was, being the first time a phonograph-graphophone was ever used as an adjunct to scientific discussion. The possibilities of this wonderful instrument are only to be surmised. It requires the imagination of an Edward Bellamy to write of an approaching phonographic age, which he has recently done in "With the Eyes Shut," in which phonographic literature is introduced on all railroads to prevent injury to the eyes in reading ordinary literature in the bad light and jolting cars. Mechanical devices announce, by means of the phonograph, the names of the different stations. He describes a not impossible experience, when hotels shall have been supplied with clocks fitted with phonographic attachments, in the following language:

"I was startled in my dreams by the voice of a young woman who could not have been standing more than ten feet from my bed. If the tones of her voice were any guide, she was not only a young woman, but a very charming one. 'My dear sir,' she had said, 'you may possibly be interested in knowing that it now wants just a quarter of three.' "

By the introduction of the phonograph-graphophone in our meetings, it is possible to add materially to their interest. Since the idea of inviting individuals, many times resident widely distant from the meeting-place, has been adopted, it is often a burden to attend in person. This can now be wholly obviated by the aid of the phonograph, which will no doubt be largely used, and every society can rent an instrument and reproduce for the delectation of its members the dulcet tones of New York's orator, the fire of Chicago's leader, or the fatherly admonitions of the patriarch in dentistry. Amusing incidents could be recorded for the edification of those not present. What would we not give for an exact record of the tilt-at-arms between "the member" from Texas and some of the brethren at Louisville summer before last, especially his laconic reply to the query of why he used sulphur in the treatment of some oral disease,—"To kill the bugs." Then again, the exact words and accents of the fathers in dentistry, who are so rapidly passing away, could be stored up and each society have its library of registered phonograms. Exchange copies could be had and the characteristics of speech and voice of those whom we know by name could be introduced to us in person, and in after years, when otherwise nothing but their writings remained, we could take down the special cylinder and spend a few minutes in private conversation with them, thus getting, as it were, a special interview, which always puts the individual on better terms with the writer than anything else.

We prize the autographs of celebrated men; how much more so would we cherish their phonograms!

